# Isolation of mixed subtypes of influenza A virus from a bald eagle *(Haliaeetus leucocephalus)*

**DOI:** 10.1186/1743-422X-7-174

**Published:** 2010-07-28

**Authors:** Sagar M Goyal, Naresh Jindal, Yogesh Chander, Muthanan A Ramakrishnan, Patrick T Redig, Srinand Sreevatsan

**Affiliations:** 1 Department of Veterinary Population Medicine, University of Minnesota, College of Veterinary Medicine, 1333 Gortner Avenue, Saint Paul, MN 55108, USA

## Abstract

From April 2007 to March 2008, cloacal swabs were obtained from 246 casualty raptors recovered by various wildlife rehabilitation centers in the United States. The swabs were placed in a virus transport medium and transported to the laboratory on ice packs. At the laboratory, the samples were pooled with each pool consisting of five samples. All pools (n = 50) were screened for the presence of avian influenza virus (AIV) using a real time reverse transcription-polymerase chain reaction (rRT-PCR); one of the pools was found positive. All five samples in this pool were tested individually by rRT-PCR; one sample from a bald eagle was found positive. This sample was inoculated in embryonated chicken eggs for virus isolation and a hemagglutinating virus was isolated. Complete genome sequencing of the isolate revealed a mixed infection with H1N1 and H2N1 subtypes. Further analysis revealed that the PB1-F2 gene sequence of H1N1 virus had the N66S virulence-associated substitution. Further studies on ecology and epidemiology of AIV in raptors are needed to help understand their role in the maintenance and evolution of AIV.

## Findings

Wild birds are considered natural reservoirs of avian influenza virus (AIV) and at least 105 species of wild birds have been reported to harbor these viruses [1]. The migratory nature of these bird populations may help disseminate AIV across countries and continents. Most of the wild birds have been reported to harbor low pathogenic avian influenza (LPAI) viruses [2-5] although highly pathogenic avian influenza (HPAI) viruses have also been isolated from some species [6]. To understand how AIV is evolving in nature, it is important to identify the AIV subtypes circulating within wild bird populations.

A number of surveillance programs have been undertaken to isolate and identify the subtypes of AIV present in wild bird species e.g., waterfowl and geese [3,7,8] with limited reports in raptors. Thus, van Borm et al [9] detected HPAI H5N1 in Thai eagles that were smuggled into Belgium and Ducatez et al [10] isolated HPAI H5N1 from vultures in Burkina Faso. Due to their carnivorous feeding habits, raptors may also play an important role in the spread of AIV. Some species of raptors often feed on ducks and geese and if these birds are carrying AIV, raptors may pick up the virus and spread it elsewhere. In this communication, we report on the isolation and characterization of two subtypes of AIV from a single bald eagle (*Haliaeetus leucocephalus*).

From April 2007 to March 2008, under an NIH funded surveillance program on avian influenza, cloacal swabs were collected from 246 casualty raptors (bald eagle-81, great horned owl-69, coopers hawk-62, turkey vulture-28, and black vulture-6) recovered by various wildlife rehabilitation centers in the U.S. The swabs were collected in brain heart infusion broth containing penicillin (500 IU/mL), streptomycin (500 μg/mL), neomycin (150 μg/mL), fungizone (1.5 μg/mL), and gentamicin (50 μg/mL). After collection, the swabs were transported on ice to the laboratory in Saint Paul by overnight shipping. At the laboratory, these samples were stored frozen at -80°C until tested (within six months of collection). The experimental plan was to thaw the samples and pool them in pools of five samples each followed by a matrix gene based real time reverse transcription-polymerase chain reaction (rRT-PCR) to detect the presence of AIV [11]. From the positive pools, individual samples were retested by rRT-PCR. All individual samples positive by rRT-PCR were subjected to virus isolation by inoculating them into 9-day-old specific-pathogen-free embryonated chicken eggs (2 eggs per sample) via the allantoic route. The inoculated eggs were incubated at 37°C for four days after which they were chilled and their allantoic fluids tested for hemagglutination (HA) using 0.5% chicken erythrocytes. RNA was extracted from HA positive samples and from a known AIV (H9N2) isolate using QIAamp viral RNA kit (Qiagen, Valencia, CA). The RNA was tested for AIV in a one-step RT-PCR using primers specific to the matrix gene of AIV [12]. Amplified PCR products were separated by gel electrophoresis and a band of 1,027 bp indicated the presence of AIV. Complete genome sequencing was undertaken using 454 pyrosequencing recently developed in our laboratory [13] and the sequences were analyzed for most closely related sequences in GenBank.

One of the 50 pools was found positive for AIV by rRT-PCR. All five cloacal samples in the positive pool were then tested individually by rRT-PCR and a bald eagle sample was positive. This sample was inoculated in embryonated chicken eggs for virus isolation and a hemagglutinating virus was isolated. Whole genome sequence of this isolate was obtained using the 454-pyrosequencing technique that enables resolution beyond what is possible by standard Sanger sequencing. Contigs of all eight segments of influenza A virus were first identified using GenAssembler software (454 Life Sciences, Roche, Branford, CT) that detected two distinct hemagglutinin subtypes - H1 and H2 (GenBank Accession nos. CY043315 - CY043330). Using standard BLAST algorithm, GenBank databases were queried for the most closely related isolates. Sequences of A/green-winged teal/Ohio/430/1987(H1N1) (CY011040) and A/mallard/Ohio/37/1986(H2N1) (CY021125) were homologous to those of the bald eagle isolate. Sequences of all eight segments from these two subtypes were used as a reference for subsequent alignments performed with GenMapper (454 Life Sciences, Roche, Branford, CT). The alignments yielded full genome sequences for two subtypes, H1N1 and H2N1. This was further confirmed by manual alignment of all contigs obtained from GenAssembler and GenMapper using Sequencher (Gene Codes Corporation, Ann Arbor, MI).

On further analysis, the matrix gene of H1N1 was found to be more closely related to A/sanderling/DE/1258/1986(H6N6). The nucleotides of HA and NA genes of H2N1 subtype had 99% and 100% identity, respectively, with those of A/mallard/Ohio/37/1986(H2N1). The nucleotide identities between each segment of the two subtypes were also determined (Table 1). One virulence-associated polymorphism in PB1-F2 (N66S) was present in the H1N1 virus (CY043317) [14].

**Table 1:** Genetic similarity between A/bald eagle/Virginia/Sg-00154/2008(mixed)) and reference strains available in GenBank (Accession date: 1 Feb 2010)

Gene segment	GenBank Accession number of Bald eagle isolate^a^	Bald eagle isolate showing the highest nucleotide identity with:		Nucleotide identity between two subtypes of bald eagle isolate (%)
		
		Virus Name (GenBank Accession number)	Percent identity	
**PB2**	CY043315	A/green-winged teal/Ohio/430/1987(H1N1) (CY011047)	99%	92
	CY043316	A/mallard duck/New York/157/1986(H3N6) (CY014871)	99%	
				
**PB1**	CY043317	A/green-winged teal/Ohio/430/1987(H1N1) (CY011046)	99%	93
	CY043318	A/mallard/Ohio/37/1986(H2N1) (CY021131)	99%	
				
**PA**	CY043319	A/green-winged teal/Ohio/430/1987(H1N1) (CY011045)	99%	89
	CY043320	A/mallard/Ohio/48/1986(H3N2) (CY020722)	99%	
				
**HA**	CY043321	A/green-winged teal/Ohio/430/1987(H1N1) (CY011040)	99%	68
	CY043322	A/mallard/Ohio/37/1986(H2N1) (CY021125)	99%	
				
**NP**	CY043323	A/green-winged teal/Ohio/430/1987(H1N1) (CY011043)	100%	95
	CY043324	A/mallard/Ohio/37/1986(H2N1) (CY021128)	99%	
				
**NA**	CY043325	A/green-winged teal/Ohio/430/1987(H1N1) (CY011042)	100%	96
	CY043326	A/mallard/Ohio/30/1986(H2N1) (CY017695)	100%	
				
**M**	CY043327	A/sanderling/DE/1258/1986(H6N6) (CY005421)	99%	97
	CY043328	A/mallard duck/New York/157/1986(H3N6) (CY014866)	99%	
				
**NS**	CY043329	A/green-winged teal/Ohio/430/1987(H1N1) (CY011044)	100%	80
	CY043330	A/mallard duck/New York/180/1986(H4N9) (CY014861)	99%	

^a ^The GenBank accession number appearing in the top row of each segment represents the H1N1 subtype sequence while that in the bottom row represents the H2N1 subtype.

All AIV subtypes (except certain H5 and H7 subtype combinations) are considered to be LPAI in nature and generally do not cause apparent disease in avian species. Even infection with HPAI H5N1 is not fatal to some waterfowl species [15]. Most of the surveillance studies [3,4,7,8] have focused on the detection of AIVs from waterfowl and shorebirds while only a few reports are available on isolation of AIV from raptors. These include reports on the isolation of H7 subtype from saker falcon [16], crested hawk eagles [9], and wild hooded vultures [10]. In addition, De Marco et al. [17] reported serologic evidence of influenza A virus exposure in diurnal raptors in a rehabilitation facility. In the winter of 2005-06, there were reports of H5N1-related mortality among raptor species in Europe and Asia, including eagle owls (*Bubo bubo*), buzzards (*Buteo lagopus*), and peregrine falcon (*Falco peregrinus*) (ProMED).

This is the first report on isolation of AIV in a Bald eagle from the U.S. The bald eagle in question was recovered from Gloucester County, Virginia in March, 2008. In general, samples from injured raptors are collected immediately after admission to the Rehabilitation Center and before mixing with raptors already residing at the Rehabilitation Center. These procedures were in place when the bald eagle was brought to the Rehabilitation Center. In addition, the raptors residing at the Rehabilitation Center were AIV negative, thus ruling out the possibility of the bald eagle acquiring AIV infection at the Rehabilitation Center.

The bald eagle, a bird of prey, is a protected and revered species in the US. In addition to fish, these birds prey on ducks and geese [18], resulting in possible exposure to AIV. The AIV subtypes obtained from raptors probably reflect the virus types present in its prey. The raptors may also be infected from domestic poultry especially in areas where dead poultry carcasses are discarded in the open or in the presence of open abattoir systems. Though we do not know the role of raptors in virus transmission to domestic poultry or wild birds at present, such a possibility does exist. Since a large pool of influenza subtypes circulates in waterfowl populations [3,7], which are preys of raptors, further studies are needed to determine the prevalence of different AIV subtypes in raptors and their impact vis-a-vis transmission to domestic poultry directly or in a chain involving raptors-waterfowl-domestic birds. Perhaps more importantly, raptors, especially eagles, may serve as sentinels for influenza viruses circulating in the environment from which they obtain their prey.

The genome sequences of this bald eagle isolate revealed a mixture of H1N1 and H2N1 subtypes. Phylogenetic analysis identified a high degree of sequence similarity of all segments of these two subtypes to influenza A viruses circulating in wild waterfowl populations suggesting that the bald eagle possibly acquired the viruses while preying on infected wild birds. Our results indicate the need to study the raptor population in detail to gain better understanding of AIV ecology and epidemiology.

## Abbreviations

AIV: avian influenza virus; HPAI: highly pathogenic avian influenza virus; LPAI: low pathogenic avian influenza virus; NIH: National Institutes of Health; rRT-PCR: real time reverse transcriptase polymerase chain reaction; RT-PCR: reverse transcriptase polymerase chain reaction

## Competing interests

The authors declare that they have no competing interests.

## Authors' contributions

PTR was responsible for collection of samples from bald eagles. SMG and SS were responsible for overall coordination and planning of the study. NJ, YC, and MAR contributed in virus isolation, RT-PCR, sequencing and sequence analysis. NJ, SMG, and SS drafted the manuscript. All authors' have read and approved final manuscript.
